# Contrast-enhanced MR microscopy of amyloid plaques in five mouse models of amyloidosis and in human Alzheimer’s disease brains

**DOI:** 10.1038/s41598-017-05285-1

**Published:** 2017-07-10

**Authors:** Clémence Dudeffant, Matthias Vandesquille, Kelly Herbert, Clément M. Garin, Sandro Alves, Véronique Blanchard, Emmanuel E. Comoy, Fanny Petit, Marc Dhenain

**Affiliations:** 1 Centre National de la Recherche Scientifique (CNRS), Université Paris-Sud, Université Paris-Saclay UMR 9199, Neurodegenerative Diseases Laboratory, F-92260 Fontenay-aux-Roses, France; 2 Commissariat à l’Energie Atomique et aux Energies Alternatives (CEA), Direction de la Recherche Fondamentale (DRF), Molecular Imaging Research Center (MIRCen), F-92260 Fontenay-aux-Roses, France; 3 INSERM U986, Université Paris-Sud, 94276 Le Kremlin-Bicêtre, France; 4 Sanofi, Translational Science Unit, Molecular Histopathology and Bioimaging, Chilly-Mazarin, France; 5 Commissariat à l’Energie Atomique et aux Energies Alternatives (CEA), Direction de la Recherche Fondamentale (DRF), Institut des Maladies Emergentes et des Therapies Innovantes (IMETI), SEPIA, 18 Route du Panorama, F-92265 Fontenay-aux-Roses, France

## Abstract

Gadolinium (Gd)-stained MRI is based on Gd contrast agent (CA) administration into the brain parenchyma. The strong signal increase induced by Gd CA can be converted into resolution enhancement to record microscopic MR images. Moreover, inhomogeneous distribution of the Gd CA in the brain improves the contrast between different tissues and provides new contrasts in MR images. Gd-stained MRI detects amyloid plaques, one of the microscopic lesions of Alzheimer’s disease (AD), in APP_SL_/PS1_M146L_ mice or in primates. Numerous transgenic mice with various plaque typologies have been developed to mimic cerebral amyloidosis and comparison of plaque detection between animal models and humans with new imaging methods is a recurrent concern. Here, we investigated detection of amyloid plaques by Gd-stained MRI in five mouse models of amyloidosis (APP_SL_/PS1_M146L_, APP/PS1_dE9_, APP23, APP_SwDI_, and 3xTg) presenting with compact, diffuse and intracellular plaques as well as in *post mortem* human-AD brains. The brains were then evaluated by histology to investigate the impact of size, compactness, and iron load of amyloid plaques on their detection by MRI. We show that Gd-stained MRI allows detection of compact amyloid plaques as small as 25 µm, independently of their iron load, in mice as well as in human-AD brains.

## Introduction

Amyloid plaques are one of the earliest hallmarks of Alzheimer’s disease (AD), occurring up to 20 years before clinical diagnosis^1^. Even if their role in AD onset is still debated, they appear as an effective biomarker of its preclinical stages. Currently, the clinical detection of amyloid plaques is based on positron emission tomography (PET) imaging with three radioactive agents recently approved by the Food and Drug Administration (FDA)^2^. However, the low spatial resolution of PET does not allow the visualization of individual plaques, and in animals, PET studies have provided controversial results^3, 4^. For example, some studies successfully detected amyloid progression in APP23^5^ and 5xFAD^6^ mice while others failed to detect signal changes related to amyloidosis^7, 8^. Other imaging modalities, such as optical imaging^9^ or two-photon imaging after craniotomy^10^, have also been developed to detect amyloid plaques in animals. As with PET, optical imaging is too low-resolution to identify individual plaques. Two-photon imaging, however, can reveal individual amyloid plaques at very high resolution (1 μm)^10^ though the field of view of the technique is limited and does not allow recording of images from the whole brain^11^.

Continuous efforts are ongoing to implement amyloid plaque detection by high-resolution magnetic resonance imaging (MRI). MRI-based monitoring of amyloid plaques can be divided into three research fields. Some studies are based on the endogenous contrast of the plaques, in both mouse models of amyloidosis^12–15^ and in humans^16, 17^. This approach is limited by a low sensitivity threshold and is strongly dependent on the iron load of the plaques which locally shortens relaxation times^18, 19^. Also, the possibility to detect plaques by MRI in human tissues on the basis of their endogenous contrast is still disputed^18, 20, 21^. Thus, MR contrast agents seem to be required to facilitate amyloid plaque detection. The first option is to use MR contrast agents specifically targeting amyloid plaques, modulating their MR signal and so increasing their contrast with the brain parenchyma^22–25^. The second option uses non-targeted gadolinium (Gd) contrast agents such as gadoterate meglumine (Dotarem®, Guerbet, France) that is administered in cerebral ventricles after stereotaxic injection^26^ or intravenously in association with a non-invasive and safe permeation of the blood-brain barrier using ultrasound^27^. With this method, called Gd-stained MRI, once the contrast agent has reached the brain, amyloid plaques appear as black spots since the hydrophilic Gd-contrast agent increases the signal of tissues surrounding the plaques but do not access their hydrophobic core^28^. As the volume of brain tissue is high compared to the volume of plaques, these agents induce a high signal increase in the brain. This can be converted into resolution enhancement to record high resolution images. Several *in vivo* studies in mice have shown that this method reveals amyloid plaques that otherwise cannot be detected by non-enhanced MRI^26, 27^. Recently, it was used to characterize longitudinally the efficacy of an anti-amyloid immunotherapy^29^. Gd-stained MRI has also been used to detect amyloid plaques in primates^30^ and to detect prion plaques in *post mortem* brain samples from Creutzfeldt-Jakob patients^31^, but it has never been used to label amyloid plaques in human-AD brains.

Numerous models of amyloidosis with different plaque typologies are used for preclinical investigations. The choice of transgenic mouse model, as well as the stage of Aβ pathology, significantly contributes to the outcome of preclinical studies. Here, we investigated the extent to which Gd-stained MRI allows detection of different types of amyloid lesions including compact, diffuse and intracellular amyloid deposits in five mouse models of amyloidosis (APP_SL_/PS1_M146L_, APP/PS1_dE9_, APP23, APP_SwDI_, 3xTg). Vascular abnormalities often co-exist with amyloid plaques in mouse models of amyloidosis as in AD patients^32^. Aβ peptide may accumulate into the vessel wall of cerebral arteries leading to cerebral amyloid angiopathy (CAA) and microhemorrhages. We also examined the capacity of Gd-stained MRI to detect these lesions. Finally, we explored the capacity of Gd-stained MRI to detect amyloid plaques in *post mortem* human-AD brains. The brain samples were then evaluated by histology to assess the impact of size, compactness, and iron load of amyloid plaques on their detection by MRI. Amyloid plaques from APP_SL_/PS1_M146L_, APP/PS1_dE9_ and human-AD brains had the most similar histological characteristics and could be detected by Gd-stained MRI. Also, we found that the key features associated to amyloid plaque detection by Gd-stained MRI are their size, compactness but not their iron load.

## Results

### Heterogeneity of amyloid plaque detection in mice

**Figure 1:**
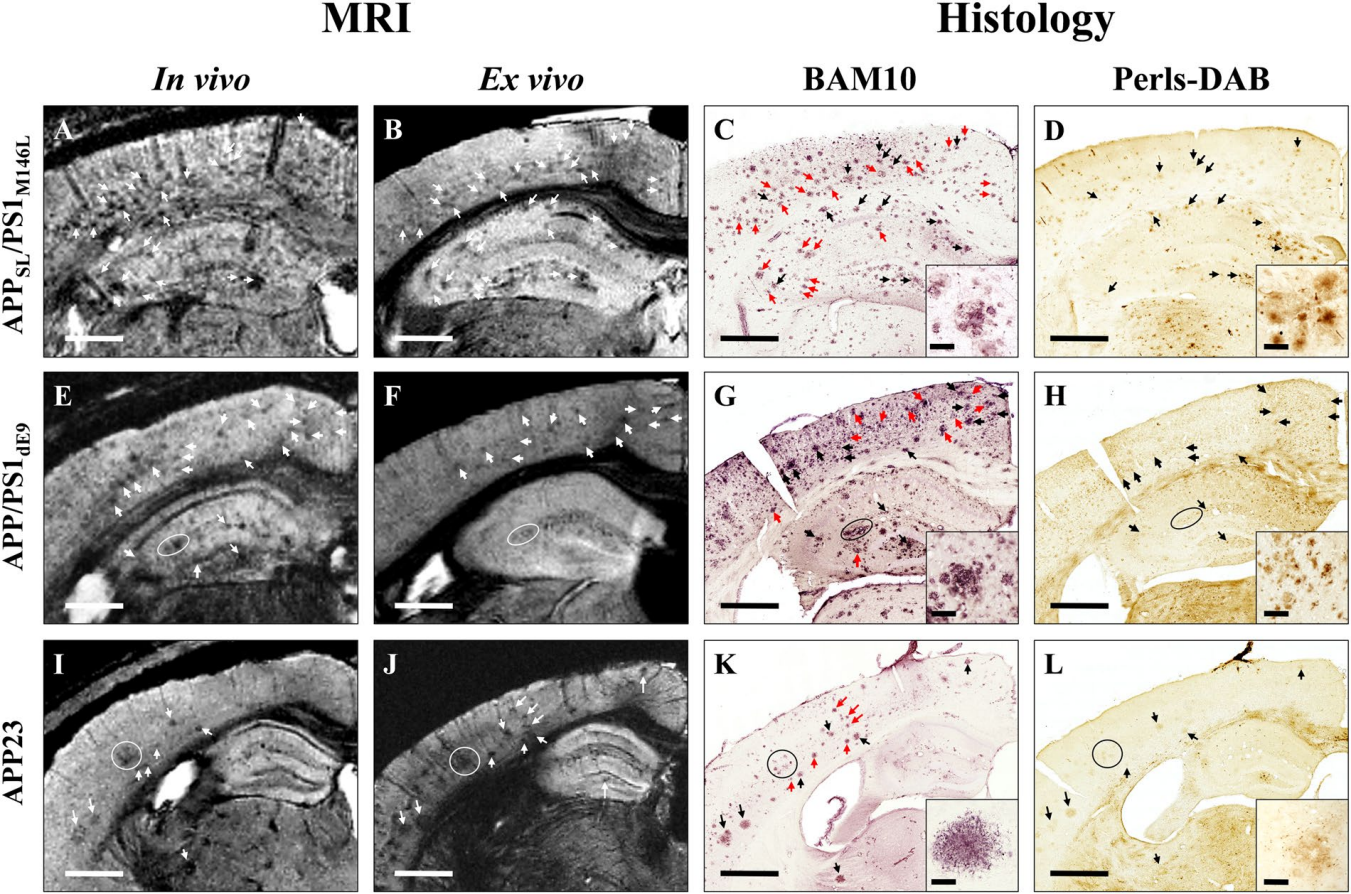
Comparison between detection of amyloid plaques by Gd-stained MRI and immunohistochemistry in APP_SL_/PS1_M146L_, APP/PS1_dE9_, and APP23 mice. Gd-stained *in vivo* (column 1) *or ex vivo* (column 2) MR images were registered with β-amyloid (BAM10, column 3) and iron-stained (Perls-DAB, column 4) histological sections in APP_SL_/PS1_M146L_ (**A**–**D**), APP/PS1_dE9_ (**E**–**H**) and APP23 (**I–L**) mice. Inserts in columns 3 and 4 display typical plaques for each strain. Hypointense spots (white arrows and circles) are visible in the *in vivo* and/or *ex vivo* MR images of APP_SL_/PS1_M146L_ (**A**,**B**), APP/PS1_dE9_ (**E**,**F**) and APP23 (**I**,**J**) mice. They can be registered with amyloid plaques (black and red arrows and circles) on BAM10-stained sections (**C**,**G**,**K**). Iron staining reveals iron deposits that can be registered with hypointense spots and amyloid plaques in APP_SL_/PS1_M146L_ (**D**), APP/PS1_dE9_ (**H**) and APP23 (**L**) mice. Some amyloid plaques containing iron (black arrows and circles) are visible on MR images. Some others are iron-free (red arrows) and are also detected by MRI indicating that iron is not necessary for MR detection. Scale bars: 500 µm for main images and 50 µm for inserts.

**Figure 2:**
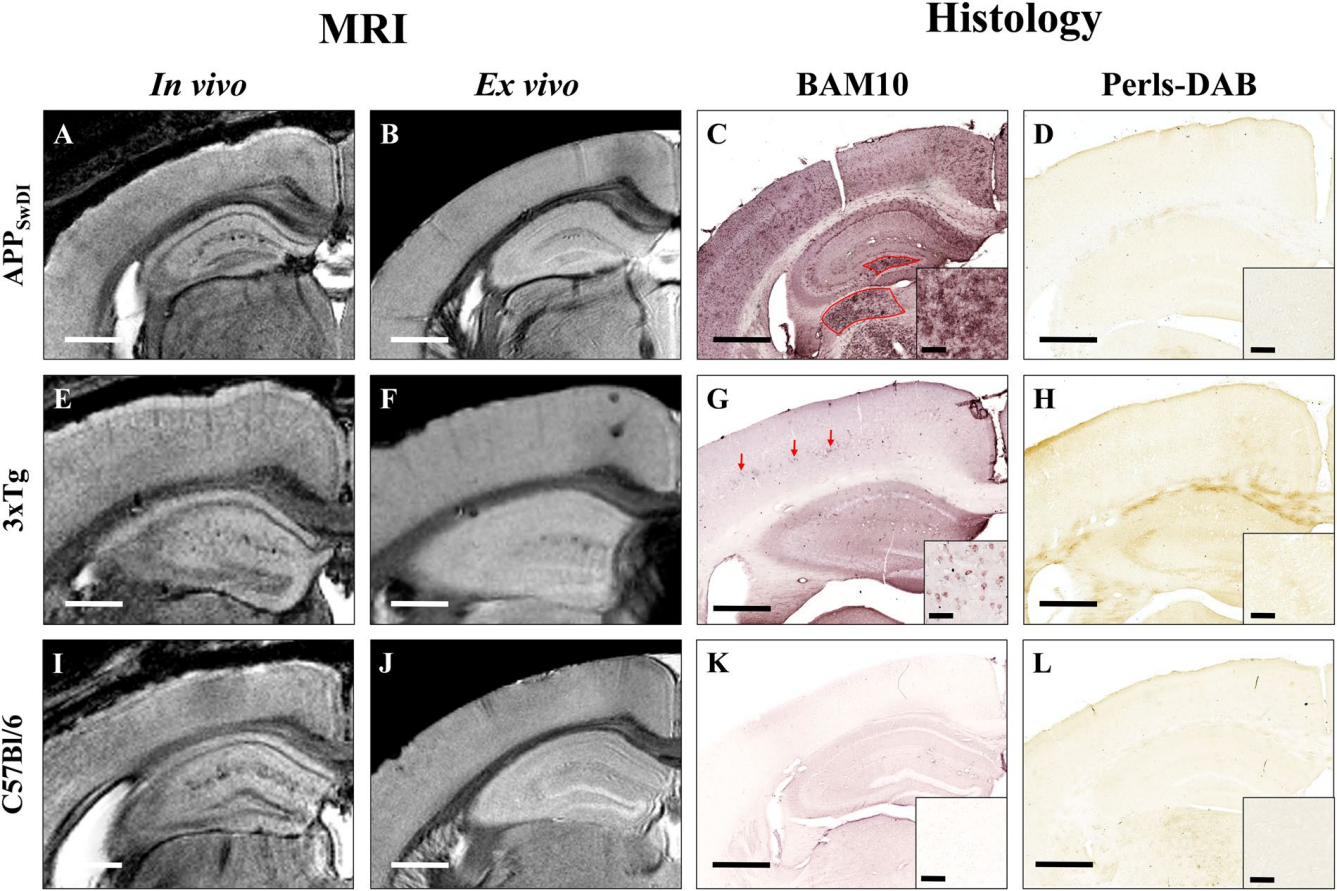
Comparison between detection of amyloid plaques by Gd-stained MRI and immunohistochemistry in APP_SwDI_, 3xTg, and C57Bl/6 amyloid-free mice. Gd-stained *in vivo* (column 1) *or ex vivo* (column 2) MR images were registered with β-amyloid (BAM10, column 3) and iron-stained (Perls-DAB, column 4) histological sections in APP_SwDI_ (**A–D**), 3xTg (**E–H**) and C57Bl/6 amyloid-free (**I–L**) mice. Inserts in columns 3 and 4 display typical plaques for each strain. MR images of APP_SwDI_ mice do not present with hypointense spots (**A**,**B**). BAM10 and iron staining show large diffuse Aβ-positive lesions (**C**, red shape) devoid of iron deposits (**D**). 3xTg mice do not present with hypointense spots on MR images (**E**,**F**). BAM10 and iron staining show intracellular Aβ deposits (**G**, red arrows) devoid of iron (**H**). In C57Bl/6 amyloid-free mice, no hypointense spots on MR images (**I**,**J**) or amyloid plaques on BAM10 sections (**K**) are detected. Scale bars: 500 µm for main images and 50 µm for inserts.

Gd-stained MRI was performed on five mouse models of amyloidosis (APP_SL_/PS1_M146L_, APP/PS1_dE9_, APP23, APP_SwDI_ and 3xTg) and C57Bl/6 amyloid-free control animals. *In vivo* MRI was acquired at a resolution of 29 × 29 × 117 µm^3^ after intracerebroventricular injection of gadoterate meglumine. *Ex vivo* MRI was recorded at a resolution of 25 × 25 × 100 µm^3^ after incubation of the brains in a Gd solution. *In vivo* and *ex vivo* MRI without Gd-staining were also performed in APP_SL_/PS1_M146L_ mice. Following *ex vivo* MRI acquisitions, brains were processed by histology to label amyloid plaques or iron (Figs 1, 2).

**Figure 3:**
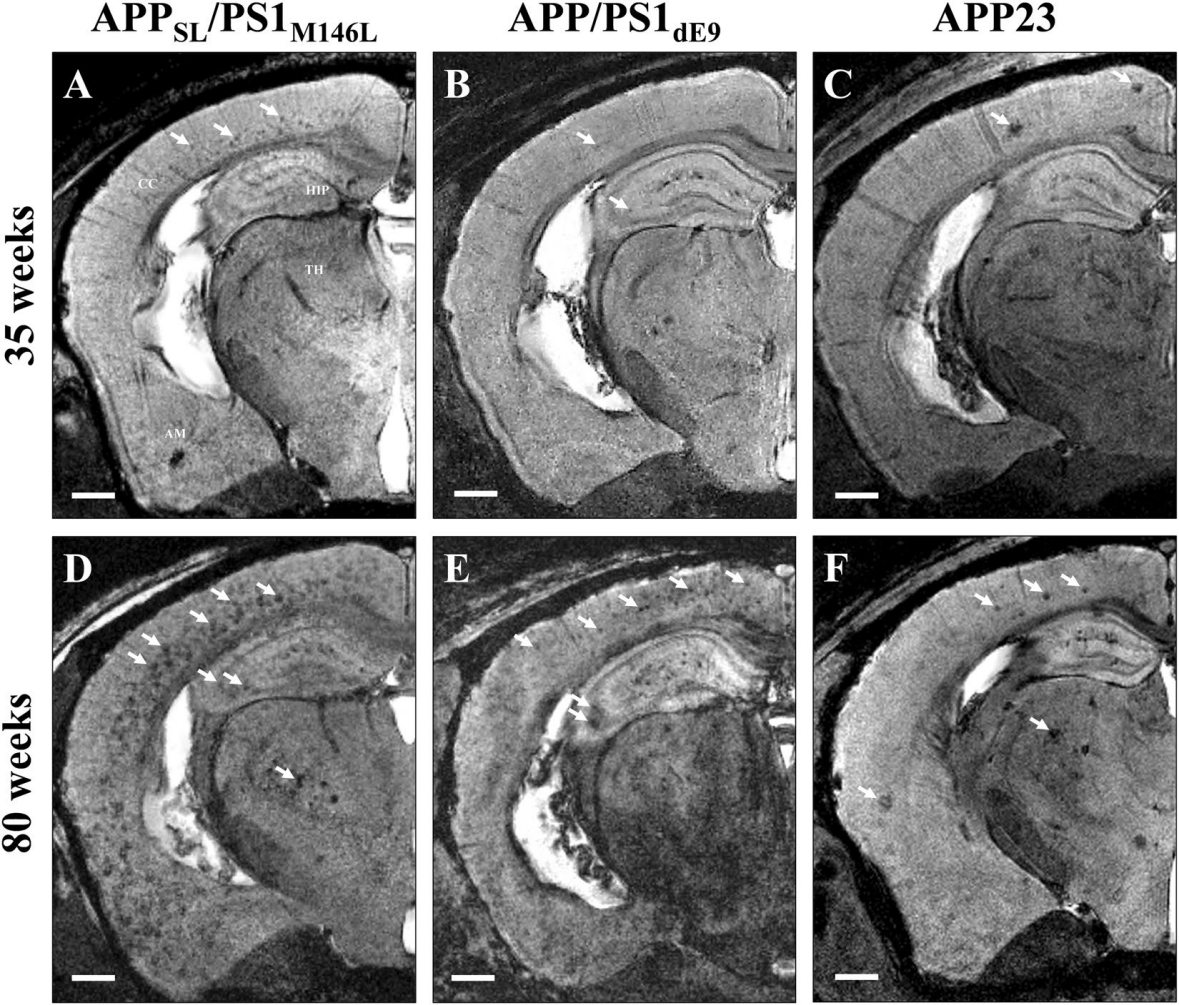
Hypointense spots on MRI sections of 35-week-old (**A–C**) and ~80-week-old (**D–F**) APP_SL_/PS1_M146L_, APP/PS1_dE9_ and APP23 mice. Hypointense spots (white arrows) are detected in the brain of 35-week-old APP_SL_/PS1_M146L_ (**A**), APPPS1_dE9_ (**B**), and APP23 (**C**) in the cerebral cortex (CC), hippocampus (HIP), thalamus (TH) and amygdala (AM). These spots increase in number and size in older animals (**D**–**F**). Scale bars: 500 µm.

**Figure 4:**
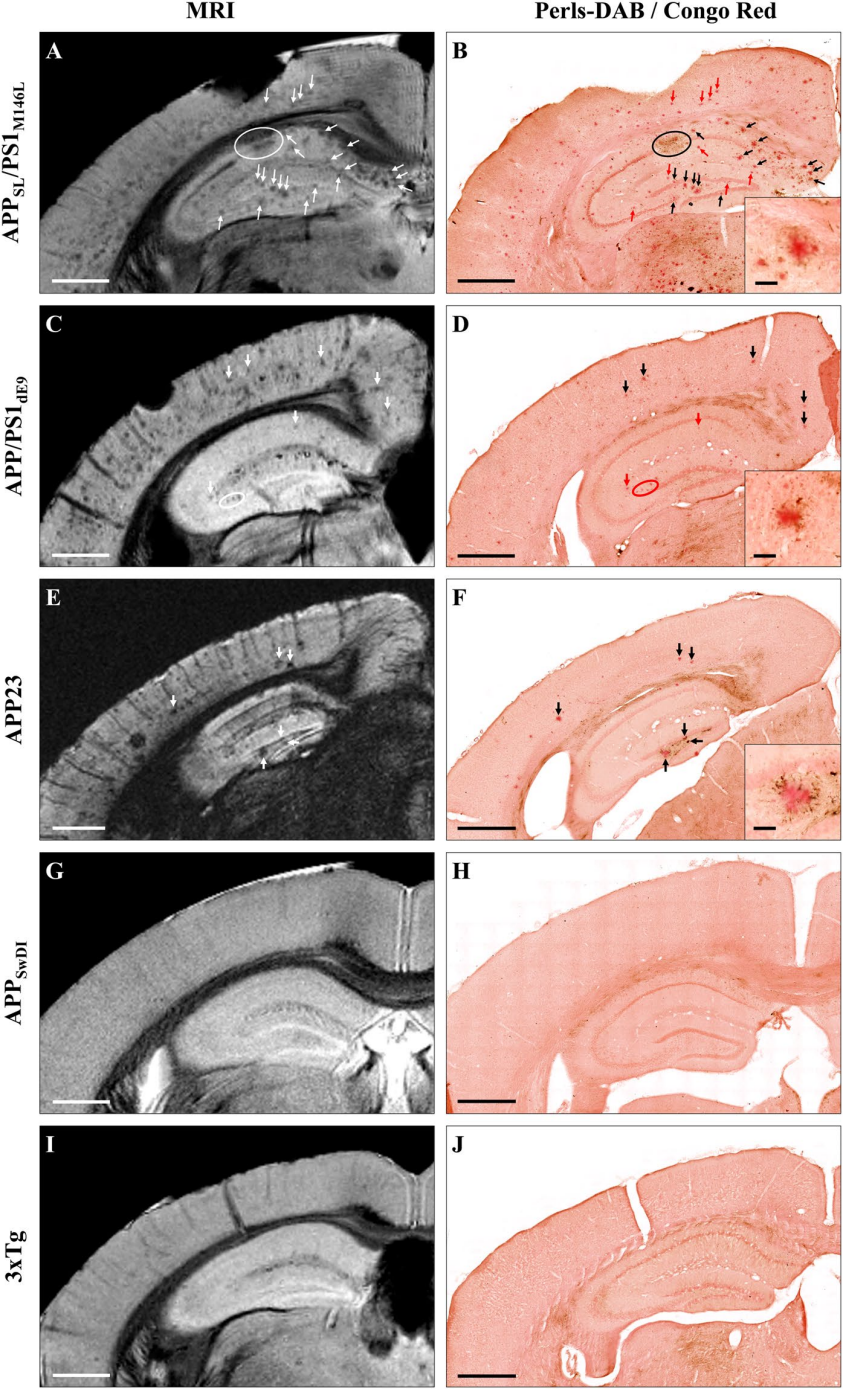
Comparison between detection of amyloid plaques by Gd-stained MRI and histological sections double-stained for amyloid and iron in five mouse strains. Gd-stained MR images (left) were registered with histological sections double-stained for β-amyloid (Congo red) and iron (Perls-DAB) (right) in APP_SL_/PS1_M146L_ (**A**,**B**), APP/PS1_dE9_ (**C**,**D**), APP23 (**E**,**F**), APP_SwDI_ (**G**,**H**) and 3xTg (**I**,**J**) mice. Hypointense spots (white arrows and circles) were visible in the MR images of APP_SL_/PS1_M146L_ (**A**), APP/PS1_dE9_ (**C**) and APP23 (**E**) mice. They could be registered with congophilic amyloid plaques on histological sections (**B**,**D**,**F**, black and red arrows and circles). No congophilic plaques were detected in APP_SwDI_ (**H**) and 3xTg (**J**) mice. Iron staining revealed iron deposits that co-localize with amyloid plaques (black arrows and circles) in APP_SL_/PS1_M146L_ (**B**), APP/PS1_dE9_ (**D**) and APP23 (**F**) mice. Some other plaques were iron-free (red arrows and circles) and were also detectable by MRI indicating that iron is not necessary for their detection. No iron accumulation was observed in APP_SwDI_ (**H**) and 3xTg (**J**) mice. Scale bars: 500 µm for main images and 50 µm for inserts.

As already reported^26^, Gd-staining increased the signal-to-noise ratio in MR images (Suppl. Fig. S1). This protocol revealed several hypointense spots that could not be detected without contrast agent (Suppl. Fig. S1). These spots were mainly found in the cerebral cortex, hippocampus, thalamus and amygdala (Figs 1–3) on *in vivo* or *ex vivo* images of APP_SL_/PS1_M146L_ (Fig. 1A,B, Suppl. Fig. S1), APP/PS1_dE9_ (Fig. 1E,F) and APP23 (Fig. 1I,J) mice. Figure 3 focuses on images of 35 and 80-week-old animals and shows the increased spot density with age. In the youngest animals, hypointense spots were mainly visible in APP_SL_/PS1_M146L_ mice (Fig. 3A) although discrete spots could be detected in the two other strains (Fig. 3B,C). In the oldest animals, the density of hypointense spots was highest in APP_SL_/PS1_M146L_ mice (Fig. 3D) although APP23 mice displayed the largest spots (Fig. 3F). Hypointense spots observed by MRI were identified as amyloid deposits by co-registering MR images with Aβ-stained histological sections (Figs 1, 2). APP_SL_/PS1_M146L_, APP/PS1_dE9_ and APP23 exhibited mainly compact plaques with a dense β-amyloid core (Fig. 1, inserts in C, G, and K). Plaques from APP23 mice were the largest but were less numerous than those of the other two strains. The smallest plaques that could be detected by *in vivo* MRI measured 36 µm, 37 µm and 46 µm for the APP_SL_/PS1_M146L_, APP/PS1_dE9_ and APP23 mice, respectively. On *ex vivo* images, the detection thresholds were 36 µm, 30 µm and 49 µm for the APP_SL_/PS1_M146L_, APP/PS1_dE9_ and APP23 mice, respectively. Iron deposition was also evaluated for each mouse strain either by using Perls-DAB staining alone (Figs 1, 2) or a double staining based on Perls-DAB and Congo red (Fig. 4). APP_SL_/PS1_M146L_ and APP/PS1_dE9_ mice displayed high focal iron accumulations while in APP23 mice iron staining was weak (Figs 1, 4). Registration between MR images and histological sections showed that, some amyloid plaques seen by MRI corresponded with iron deposits (Figs 1, 4, black arrows and circles), while some others could not easily be matched with iron-positive elements (Figs 1, 4, red arrows and circles). This suggests that iron is not mandatory for amyloid plaque detection after Gd-staining.

**Figure 5:**
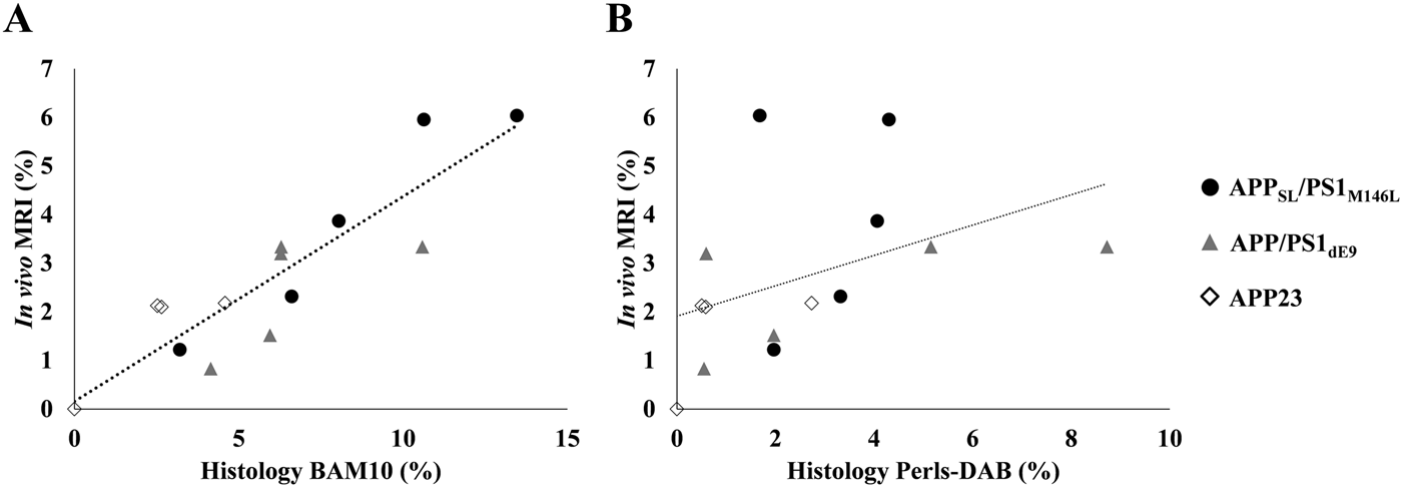
Relationships between amyloid load quantified from *in vivo* Gd-stained MR images, and from amyloid-stained histological sections (BAM10) (**A**) or from iron-stained histological sections (Perls-DAB) (**B**). Amyloid load measured from Gd-stained MRI showed a correlation with BAM10 staining (R^2^ = 0.82; p < 0.01) but was not correlated with Perls-DAB staining (R^2^ = 0.35; p > 0.05).

**Figure 6:**
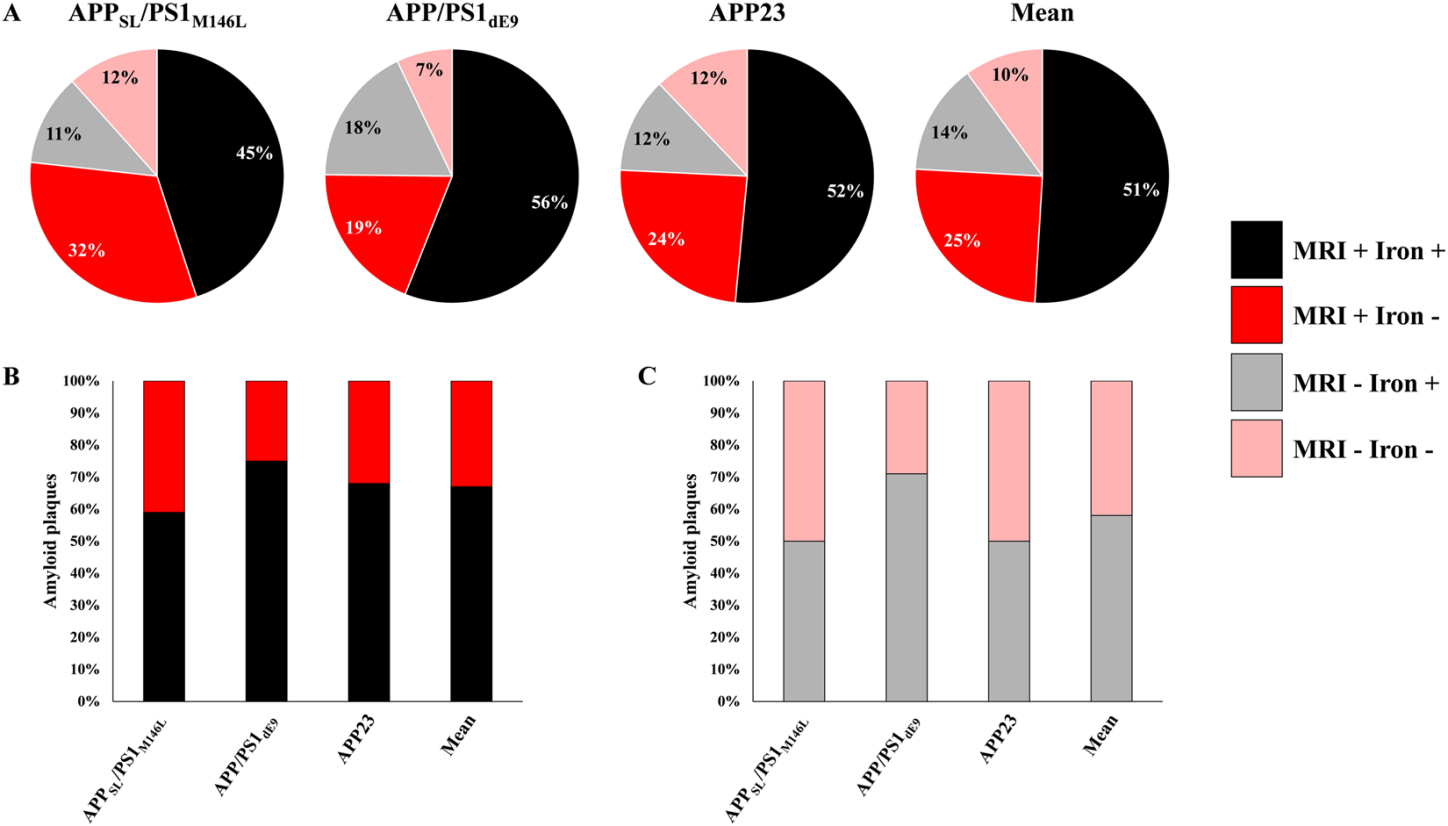
Detectability of amyloid plaques by Gd-stained MRI according to their iron content. Cortical amyloid plaques with a diameter ≥36 µm were categorized into one of the following four categories: iron-positive plaques detected by MRI (MRI + Iron +, black), iron-negative plaques detected by MRI (MRI + Iron −, red), iron-positive plaques not detected by MRI (MRI − Iron+, grey) and iron-negative plaques not detected by MRI (MRI − Iron −, pink) in APP_SL_/PS1_M146L_, APP/PS1_dE9_ and APP23 mice (**A**). Among amyloid plaques detected by Gd-stained MRI, 59%, 75% and 68% contained iron in APP_SL_/PS1_M146L_, APP/PS1_dE9_ and APP23 mice, respectively (**B**). Among amyloid plaques not detected by Gd-stained MRI, 50%, 71% and 50% contained iron in APP_SL_/PS1_M146L_, APP/PS1_dE9_ and APP23 mice, respectively (**C**).

Correlative studies were performed to further evaluate relationships between the loads of hypointense spots detected by *in vivo* MRI, amyloid plaques and iron deposits in animals in which amyloid plaques could be detected *in vivo* (APP_SL_/PS1_M146L_, APP/PS1_dE9_ and APP23). We found a significant correlation between the hypointense spots load and the amyloid load (R^2^ = 0.82; p < 0.01, Fig. 5A), but no correlation between the hypointense spots load and the iron deposits load (R^2^ = 0.35, p > 0.05, Fig. 5B). We then further evaluated the proportion of iron-positive plaques detected by Gd-stained MRI. Amyloid plaques bigger than the detection threshold (≥36 µm) were categorized into one of the following four categories: iron-positive plaques detected by MRI, iron-negative plaques detected by MRI, iron-positive plaques not detected by MRI and iron-negative plaques not detected by MRI (Fig. 6). Seventy-six percent of cortical amyloid plaques seen on histological sections were detected by Gd-stained MRI (Fig. 6A). Among these, 67% were iron-positive and 33% were iron-negative (Fig. 6B). Few differences were observed between the APP_SL_/PS1_M146L_, APP/PS1_dE9_ and APP23 mice (Fig. 6). 24% of amyloid plaques seen on histological sections were not detected by Gd-stained MRI (Fig. 6A). We cannot exclude that this lack of detection was related to an imperfect registration between MR images and histological sections because of their different thicknesses (117 and 40 µm, respectively) and because of partial volume effects. Among these 24% of amyloid plaques not detected by Gd-stained MRI, 58% were iron-positive and 42% were iron-negative (Fig. 6C).

In APP_SwDI_ mice, hypointense spots were never detected either on *in vivo* or *ex vivo* images (Fig. 2A,B). On histological sections, these mice displayed large, diffuse and poorly circumscribed plaques (Fig. 2C, red shape, Fig. 4H) and did not display any obvious iron deposits at the level of the plaques (Figs 2D, 4H).

In 3xTg mice, hypointense spots were not detected in most brain regions (Fig. 2E,F). In 3xTg mice younger than 70 weeks, amyloid deposits were mainly intracellular and measured less than 20 µm (Fig. 2G, red arrows). These intracellular deposits were not detected by Gd-stained MRI. Perls-DAB staining did not show iron accumulation within these deposits (Figs 2H, 4J). In older animals, a strong extracellular amyloidosis was observed by histology in most of the brain areas, but only plaques of the subiculum were detected by Gd-stained MRI. These subicular plaques were congophilic and iron-positive while plaques from other brain regions, not detected by MRI, were diffuse and iron-negative (Suppl. Fig. S2).

Control animals did not display any hypointense spots on MR sections (Fig. 2I,J), amyloid plaques (Fig. 2K) or focal accumulations of iron (Fig. 2L) on histological sections.

### Visualization of CAA and cerebral microhemorrhages by Gd-stained MRI

**Figure 7:**
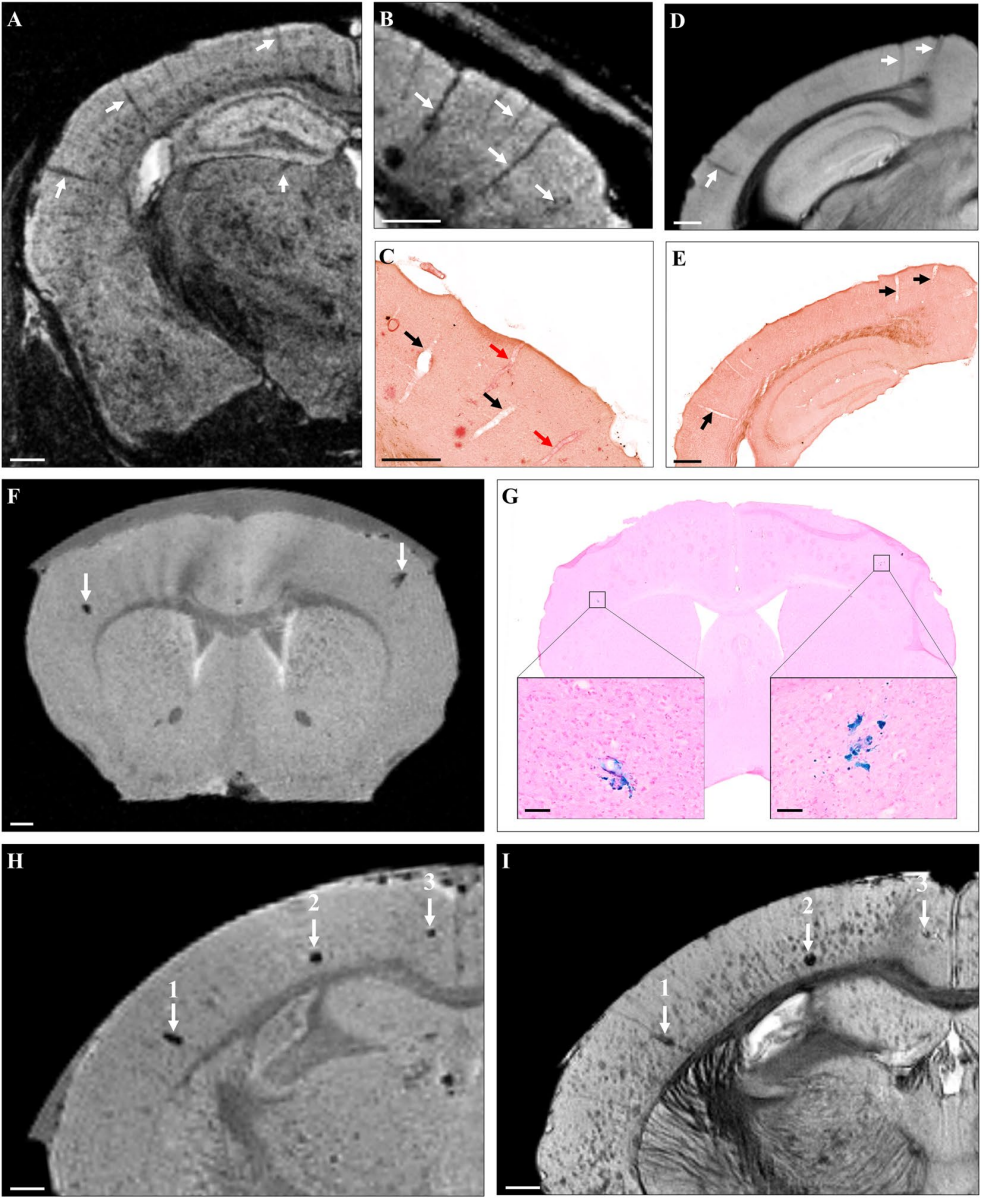
Visualization of CAA and cerebral microhemorrhages by Gd-stained MRI. Linear hypointense elements corresponding to blood vessels were detected on MR images of mouse models of amyloidosis (**A–C**, arrows). Registration between MR images (**B**) and histological sections (**C**) showed that some blood vessels seen on MRI were matched with CAA-positive vessels (red arrows) while some others were matched with CAA-negative vessels (black arrows). MR images of C57Bl/6 amyloid-free mice showed similar hypointensities (**D**, arrows) that were matched with blood vessels (**E**, arrows) confirming that CAA was not responsible of the hypointense nature of blood vessels by Gd-stained MRI. Sparse focal signal attenuations were observed on MR images before Gd-staining (**F**). They could be registered with microhemorrhages on Perls’ stained histological sections (**G**, boxes). Microhemorrhages easily seen on MRI before Gd-staining (**H**, arrows) could be matched with some hypointense spots on Gd-stained MRI (I, arrows). Large microhemorrhages (1, 2) were easily distinguished from amyloid plaques on MR images but small microbleeds (3) and amyloid plaques were similar in appearance. Scale bars: 500 µm (**A–F**,**H**,**I**) and 100 µm (**G**).

CAA and microhemorrhages are often detected in mouse models of amyloidosis. We thus evaluated whether they could be visualized by Gd-stained MRI. Blood vessels were associated with linear or punctuate hypointensities on *in vivo* and *ex vivo* Gd-stained MR images of the five studied models (Fig. 7A–C) but also of amyloid-free control animals (Fig. 7D,E). Registration between MR images and histological sections showed that the hypointense nature of blood vessels was not related to the presence of amyloid angiopathy (Fig. 7B,C).

To evaluate the appearance of microhemorrhages on Gd-stained MR images, we compared MRI recorded before and after Gd-staining. As previously reported^33–35^, on non-stained MRI, microhemorrhages were visible as rare hypointense spots (Fig. 7F,G). After Gd-staining, microhemorrhages identified in pre-contrast images remained detected in addition to amyloid plaques. Differentiation between these two lesions could be made on the basis of their size and large microbleeds were the only ones that could easily be differentiated from plaques (Fig. 7H,I). Signal from smaller microbleeds was close to that of plaques (Fig. 7H,I).

### Amyloid plaque detection in human-AD brains

**Figure 8:**
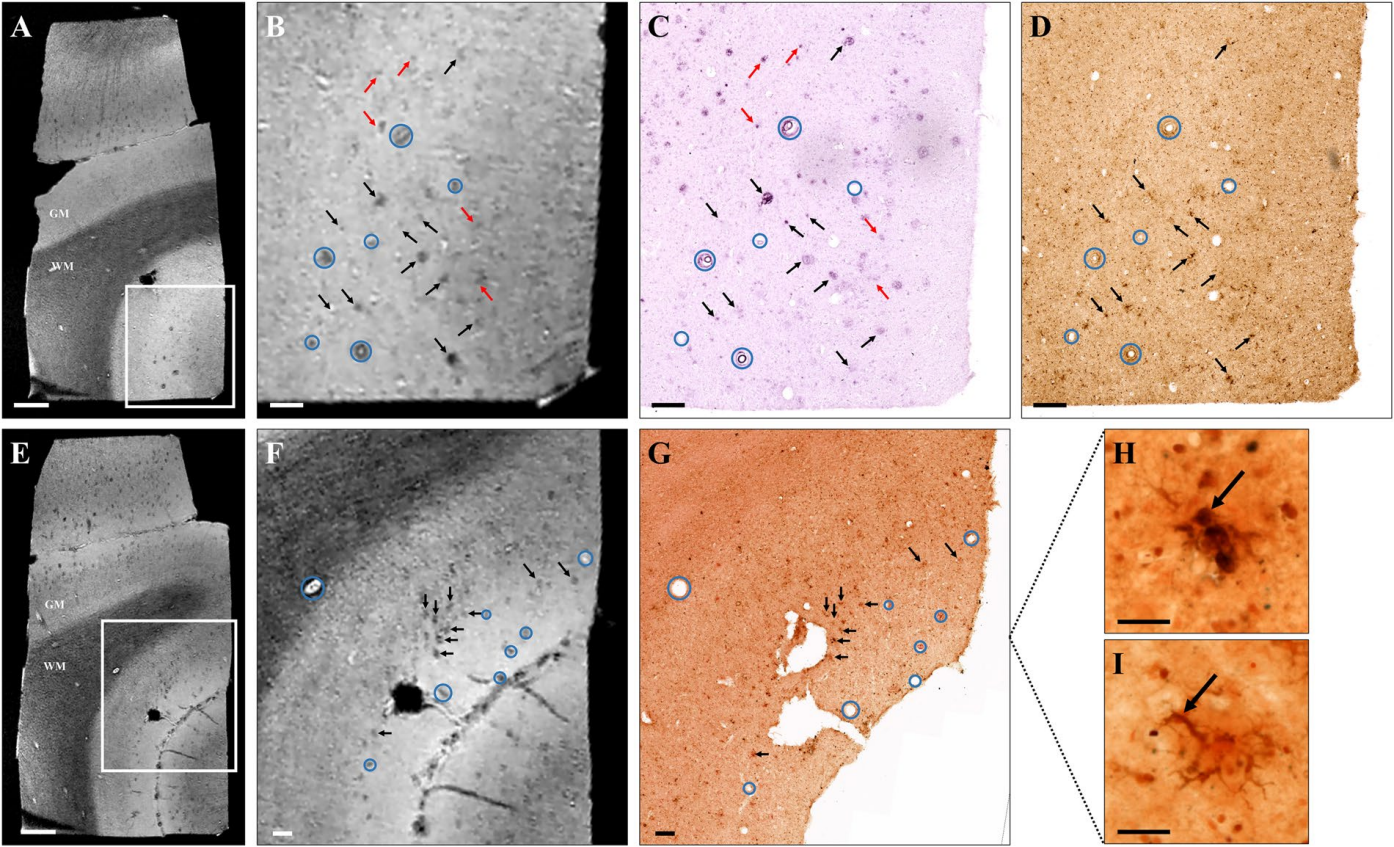
Amyloid plaque detection by Gd-stained MRI in human Alzheimer’s disease brains. Gd-stained *post mortem* MR images (**A**,**B**,**E**,**F**) were registered with histological sections stained for β-amyloid (BAM10, **C**), iron (Perls-DAB, **D**) or double-stained for β-amyloid and iron (Congo red/Perls-DAB, **G**). Grey and white matter (GM and WM respectively) could easily be identified in Gd-stained MRI (**A**,**B**,**E**,**F**). Numerous hypointense spots were visible in the grey matter of AD patients (**A**,**B**,**E**,**F**). Slices were registered according to orientation and landmarks such as blood vessels (blue circles). Most of the hypointense spots (black and red arrows) seen on MR images (**A**,**B**,**E**,**F**) can be registered with β-amyloid lesions (black and red arrows) on BAM10 (**C**) and Congo-red/Perls-DAB (**G**) stained histological sections. Iron staining (Perls-DAB) revealed iron deposits that co-localize with amyloid plaques (**D**,**G**, black arrows) in most of the human plaques. Some other plaques were iron-free (**C**,**D**, red arrows) and were also detected by MRI indicating that iron is not necessary for detection. Perls-DAB-stained sections demonstrate two types of iron accumulation near amyloid plaques (**H**,**I**): punctate (**H**) or intracellular deposits (**I**). Scale bars: 1000 µm (**A**,**E**), 200 µm (**B–D**,**F**,**G**) and 50 µm (**H**,**I**).

Brain samples from three AD patients were imaged by *ex vivo* Gd-stained MRI. Hypointense spots were detected in the cortex but not in the white matter of these patients (Fig. 8A,B,E,F). Registration between MRI and histological sections showed that most of the hypointense spots seen on MR images corresponded to amyloid plaques (Fig. 8B,C,F,G, black and red arrows) or blood vessels (Fig. 8B,C,F,G, blue circles). Hypointense spots within blood vessels can be explained by the presence of blood within the vessels in non-perfused *post mortem* samples. On histological sections, amyloid plaques measured 10 to 200 µm with most plaques smaller than 25 µm. The smallest plaques that could be detected by MRI measured 25 µm.

Finally, Perls-DAB staining revealed iron deposition in most of the human plaques detected by MRI (Fig. 8B–D,F,G, black arrows). However, as in mice, some iron-free plaques were detected by MRI (Fig. 8B–D, red arrows). Double labelling of histological sections with Perls-DAB staining and Congo red revealed two types of iron deposits associated with plaques, *i*.*e*. some punctate accumulations in the entire plaques (Fig. 8H) and ramified intracellular accumulations surrounding the plaques (Fig. 8I).

## Discussion

We compared amyloid lesions in five transgenic mouse models of amyloidosis as well as in human-AD brain samples and explored the ability of Gd-stained MRI to detect amyloid plaques in these brains. Detection of amyloid plaques by Gd-stained MRI was strikingly different among the various models used in this study and in humans. Amyloid plaques were detected *in vivo* and *ex vivo* in APP_SL_/PS1_M146L_, APP/PS1_dE9_, APP23 and to a lesser extent in 3xTg mice but never in APP_SwDI_ mice. In human-AD brains, amyloid plaques could be detected by *post mortem* Gd-stained MRI.

Our histological evaluation showed that APP_SL_/PS1_M146L_, APP/PS1_dE9_ and APP23 mice displayed mainly compact plaques. Similar lesions were the most frequently found in the human-AD brain samples studied. Striking differences were observed in the two other strains. Diffuse plaques were detected in APP_SwDI_ mice, whereas intracellular amyloid deposits were found in the 3xTg mice. Intracellular amyloid plaques have been observed in humans^36, 37^, but are not the most widely reported as they might occur in early stages of AD^38^. Regarding iron, we detected iron deposits in association with the amyloid plaques of APP_SL_/PS1_M146L_, APP/PS1_dE9_, APP23, 3xTg mice and of humans. Different iron loads were found in the different models, which is consistent with data from other studies. Jack *et al*. reported strong iron deposits in the plaques of APP_S(i.e. Tg2576)_/PS1_M146L_ mice^39^ while Meadowcroft *et al*. reported a very reduced iron load in the plaques of APP_S(i.e. Tg2576)_/PS1_A246E_ mice^18^. In humans, some studies reported a close relationship between amyloid plaques and iron depositions^18^ while some others suggested high or low iron levels in amyloid plaques even within the same subject^40^. In fact, the relationship between amyloid plaques and iron depositions in humans is still not consensual^41^. Our study supports the presence of iron in some but not all amyloid plaques. Moreover, the shape of the iron deposits is highly variable with focal deposits in mice, while in humans we detected punctate iron accumulations in the entire plaques as well as ramified accumulations surrounding the plaques.

One of the main purposes of our study was to compare MRI and histological characteristics of amyloid plaques in different mouse strains to investigate the critical parameters required for Gd-stained detection of amyloid plaques. The size of amyloid plaques is an important factor that influences their MR detection. Amyloid plaque detection was more efficient in the strains having the largest plaques as observed in APP_SL_/PS1_M146L_, APP/PS1_dE9_ or APP23 with detection thresholds of 36 and 30 µm for *in vivo* and *ex vivo* images, respectively, which corresponds approximately to 1.2 times the voxel size. This threshold can explain the poor detection achieved in the 3xTg mice. In this strain, only the largest plaques could be detected, while most of the amyloid deposits that were intracellular and smaller than 20 µm were not seen by MRI. In human-AD brains, the minimum visible plaque size was similar to that in mice (*i*.*e*. 25 µm for human-AD and 30 µm for mice).

Our study also showed that the compactness of amyloid plaques seemingly impacts their detection by the Gd-staining procedure. Amyloid plaques were detected in models with compact plaques, but not in models with large diffuse Aβ deposits (*i*.*e*. APP_SwDI_ or 3xTg mice). The mechanism of contrast enhancement is assumed to be due to the hydrophobic property of amyloid plaques that limits the penetration of the contrast agent within them. Because this hydrophobicity is related to the concentration of the amyloid peptide, diffuse deposits are probably less prone to be revealed by Gd-stained MRI than compact plaques. Interestingly, previous studies have shown that diffuse deposits are not detected by MRI without contrast agents in mice^42^ or in human brains^43^. Thus, it seems that Gd-stained MRI does not improve the ability to detect diffuse plaques as compared to contrast-agent free MRI.

Finally, our study showed that iron accumulation is not necessary for plaque detection after Gd-staining. Indeed, in mice, 33% of the amyloid plaques detected with Gd-stained MRI contained little or no iron. Also, in human-AD brain samples, although most of the plaques detected by Gd-stained MRI contained iron deposits, some plaques devoid of iron were detected. In *ex vivo* experiments without contrast agents, previous studies reported that in some transgenic mice (*i*.*e*. APP_S(i.e. Tg2576)_/PS1_A246E_ mice and APP_S(i.e. Tg2576)_/PS1_M146L_), plaques that do not contain iron can be detected^18, 42^. However, in most *in vivo* studies without contrast agent, iron is considered as critical for plaque detection^14, 15, 39^. Since iron accumulation in plaques is age-dependent and variable according to brain region, one of the advantages of Gd-stained MRI is to allow plaque detection without depending on iron accumulation which is a covariate related to aging and not to plaque load.

We then examined the capacity of Gd-stained MRI to detect microhemorrhages and CAA. Microhemorrhages are often associated with aging and amyloid pathology in mice and in humans^32^. These lesions are hypointense on MR images^33–35^, and could thus be misinterpreted as amyloid plaques on Gd-stained MR images. We showed that large microbleeds are distinguishable from amyloid plaques based on their superior size. Small microbleeds are the only lesions that could be confounded with amyloid plaques. Their number is however low compared to that of plaques, and exclusion of these lesions is always possible by recording T2* or susceptibility-weighted MR images before Gd-staining. Regarding CAA, we observed that blood vessels appeared as hypointense structures by *in vivo* MRI owing to the paramagnetic properties of blood, independently of the presence of CAA. On *post mortem* MR images, the hypointense nature of blood vessels could reflect the presence of Aβ peptide in the vessel wall but also a small amount of blood trapped in the vessels. Gd-stained MRI is thus not appropriate to detect amyloid angiopathy.

In this article, we also showed for the first time that Gd-stained MRI is able to detect amyloid plaques in human-AD brain tissues. Several significant technical barriers must be solved for this method to become suitable for use in living human subjects, including the ability to administer the contrast agent in the brain as well as resolution and imaging time considerations. The aim of this article is not to propose immediate solutions for these issues and we acknowledge that today the application of Gd-stained MRI as a routine method can be envisioned only in animal models. Gd-stained MRI can be repeated over the lifetime of animals and individual plaques can be followed-up longitudinally^26^. This method can thus be used to study amyloid biology or anti-amyloid therapies^29^. Here, we showed that among the models used in our study, APP_SL_/PS1_M146L_, APP/PS1_dE9_ and APP23 are the models of choice to apply Gd-stained MRI and amyloid plaque detection in these models provides similar results to those obtained with human brain samples.

In conclusion, this study clearly highlights differences among amyloid plaques found in different mouse models of amyloidosis, and provides a better understanding of the origin of contrast induced by amyloid plaques in Gd-stained MRI. We also showed that Gd-stained MRI can be used to detect amyloid lesions in models with large compact amyloid plaques such as APP_SL_/PS1_M146L_, APP/PS1_dE9_ or APP23 mice independently of their iron load, and suggest that detection of amyloid plaques by Gd-stained MRI in APP_SL_/PS1_M146L_ and APP/PS1_dE9_ is the most similar to that in human-AD brains.

## Material and Methods

### Animals

We selected male mice from five transgenic strains presenting with compact amyloid plaques (APP_SL_/PS1_M146L_ (n = 6), APP/PS1_dE9_ (n = 6) and APP23 (n = 4)), diffuse amyloid plaques (APP_SwDI_ (n = 6)) and intracellular amyloid deposits (3xTg (n = 7)). C57Bl/6 amyloid-free mice (n = 2) were used as controls. *In vivo* and *ex vivo* Gd-stained MR images from brains of these animals were recorded to detect amyloid plaques. Two APP_SL_/PS1_M146L_ were also imaged *in vivo* and *ex vivo* before Gd-staining. Ages of the animals were selected to image the youngest animals with already well-established lesions (based on preliminary histological studies and on published pathophysiological characteristics of each strain) and the oldest animals available in our colony (approximately 100-week-old animals). A cohort of 75 week-old APP_SL_/PS1M_146L_ mice (n = 5) was further evaluated to study microhemorrhage detection. Their brains were studied before and after Gd-staining. An overview of the selected strains is presented below.

APP_SL_/PS1_M146L_ mice co-express human APP with Swedish double mutation (KM670/671NL) and London mutation (V717I) and human presenilin 1 (PS1) with M146L mutation under the control of a neuron-specific Thy1 promoter. This leads to a 3-fold higher expression of the human APP transgene than endogenous murine APP. These mice develop dense-cored amyloid plaques that reach a significant level in the neocortex and in the hippocampus at ~26 weeks^44^. These plaques have already been widely evaluated with Gd-stained MRI^26, 27, 29^ and they can be detected in ~26-week-old animals^26^. These mice were imaged from 24 to 78 weeks (n = 6 and 5 for amyloid plaque and microhemorrhage detection, respectively).

APP/PS1_dE9_ mice co-express human APP with the Swedish double mutation (KM670/671NL) and human PS1 deleted in exon 9 under the control of the mouse prion protein promoter. Significant amyloid plaque burden is seen in the hippocampus and cortex by 36 weeks^45^ and increases with age^46^. These mice develop amyloid plaques with a dense core, surrounded by dystrophic neurites^45^. These mice were imaged from 35 to 126 weeks (n = 6).

APP23 mice express human APP with the Swedish double mutation (KM670/671NL) driven by the mouse Thy1.2 promoter allowing a neuron-specific expression of the transgene. This leads to a 7-fold higher expression of the human mutated APP than the endogenous murine APP^47^. APP23 mice develop a significant amyloidosis between 24 and 56 weeks^47, 48^. Then, dense-cored amyloid plaques increase in size and number with age, mainly in the neocortex and the hippocampus^47^. These mice were imaged from 39 to 77 weeks (n = 4).

APP_SwDI_ mice express human APP containing three mutations: Swedish (KM670/671NL), Dutch (E693Q) and Iowa (D694N), under the control of the mouse Thy1 promoter. These mice display mainly diffuse amyloid plaques and a significant number of plaques is reached at 52 weeks^49^. These mice were imaged from 52 to 114 weeks (n = 6).

3xTg mice express three mutated transgenes (APP_KM670/671NL_, MAPT_P301L_, and PSEN1_M146V_) to comparable levels in the same brain regions. Consequently, they display both amyloid and neurofibrillary tangle pathologies. Amyloidosis starts at 12 weeks in the neocortex and at 24 weeks in the hippocampus, before neurofibrillary tangle formation. At this age, amyloid is mainly intraneuronal. Extracellular amyloid deposits become readily evident at 52 weeks^50^. These mice were imaged from 44 to 112 weeks (n = 7).

C57Bl/6 are amyloid-free mice used as controls. These mice were imaged at 48 and 79 weeks (n = 2).

All animal experiments were conducted in accordance with the European Communities Council Directive (2010/63/UE). Animal care was in accordance with institutional guidelines and experimental procedures were approved by local ethics committees (authorization 12-062; ethics committee CETEA-CEA DSV IdF).

### Human-AD brain samples

Human *post mortem* brain samples from the cerebral cortex and adjacent white matter of three AD patients were obtained from the Gie-Neuro-CEB brain bank. This brain bank is run by a consortium of patients associations including France Alzheimer, with the support of Fondation Plan Alzheimer and IHU A-ICM. All methods using human brains were carried out in accordance with French guidelines and regulations. The informed consent forms were signed by either the patients themselves or their next of kin in their name, in accordance with French bioethical laws. The Brain Bank GIE NeuroCEB has been declared at the Ministry of Higher Education and Research and has received approval to distribute samples (agreement AC-2013-1887).

### Surgical procedure

Animals were anesthetized with a mixture of isoflurane (1–2%) and air (1 L/min). After their heads were shaved, the mice were placed on a stereotaxic frame using ear bars and a tooth bar to secure them. A heating pad maintained physiological temperature throughout the procedure. After a midline incision of the skin, the coordinates of the bregma were recorded for anterior-posterior (A/P) and lateral (L) references. The skull was bilaterally perforated with a Dremel at coordinates A/P −0.2 mm and L ± 1 mm, according to a stereotaxic atlas^51^. Blunt Hamilton syringes were used to inject gadoterate meglumine (DOTAREM®, Guerbet, Aulnay-sous-Bois, France) into the lateral ventricles at coordinate −1.75 mm relative to the surface of the dura mater. A total volume of 1 µL (0.5 mmol/mL) was injected into each side at a rate of 0.1 µL/minute. Upon completion of the injections, needles were not moved for 10 minutes to allow the diffusion of the contrast agent. Then, needles were slowly withdrawn to minimize any outflow from pressure release and the skin was then sutured back.

### *In vivo* MRI experiments

*In vivo* MRI was performed with a 7T spectrometer (Agilent, USA) interfaced with a console running VnmrJ 3.2. The spectrometer was equipped with a rodent gradient insert of 700 mT/m. A birdcage coil (RapidBiomed, GmbH, Germany) and a mouse brain surface coil (RapidBiomed, GmbH, Germany) were used for emission and reception, respectively. A high-resolution 3D-Gradient Echo sequence was used to achieve a resolution of 29 × 29 × 117 µm^3^ (matrix = 512 × 512 × 128, repetition time (TR) = 50 ms, echo time (TE) = 13 ms, flip angle = 20°, number of averages (Nex) = 2, bandwidth = 25 kHz, acquisition time = 1 h 49 min)^26^. All the MR images were recorded starting at 60 minutes after administration of the Gd contrast agent. During MRI experiments, animals were anesthetized with a mixture of isoflurane (0.75–1.5%) and carbogen (95% O_2_–5% CO_2_). Their breathing rate and their body temperature was monitored. Carbogen was used to reduce the signal coming from circulating blood^52^.

All animals were sacrificed after *in vivo* MRI experiments using a high dose of sodium pentobarbital (100 mg/kg) and then fixed with a transcardiac perfusion of 4% paraformaldehyde (PFA). The brains were then removed, immersed in 4% PFA overnight at 4 °C, and preserved in PBS 0.1 M at 4 °C until *ex vivo* MRI experiments.

### *Ex vivo* MRI experiments

Brains were incubated in a Gd solution (DOTAREM^®^ diluted to 2.5 mM in PBS) for 48 hours before MR experiments. Then, they were placed in a tight plastic tube filled with an aprotonic perfluorocarbon-based fluid (Fluorinert^®^, 3 M^™^) that provides a black background. A high-resolution 3D-Gradient Echo sequence was used to achieve a resolution of 25x25 × 100 µm^3^ (matrix = 512 × 512 × 128, TR = 40 ms, TE = 15 ms, flip angle = 20°, Nex = 2, bandwidth = 25 kHz, acquisition time = 11 h 39 min).

To detect microhemorrhages, *ex vivo* MR images were recorded to achieve a resolution of 50.8 × 50.8 × 50.8 µm^3^ (matrix = 256 × 256 × 512, TR = 40 ms, TE = 15 ms, flip angle = 20°, Nex = 8, acquisition time = 11 h 39 min).

### Histology

Brains were cryoprotected in 30% PBS-sucrose solution for 72 hours, cut into 40 µm thick coronal sections on a freezing microtome and mounted on slides (Ultrafrost, Thermo-Fisher®). Sections were stained for β-amyloid deposits (BAM10 immunohistochemistry and Congo red staining) and for iron deposits (Perls-DAB staining).

For BAM10 immunohistochemistry, sections were first rinsed in PBS 0.1 M and then in 30% hydrogen peroxide (H_2_O_2_). Then, they were pretreated with 0.2% octylphenol ethylene oxide condensate (Triton X-100™, Sigma-Aldrich®). After this pretreatment, they were incubated with an anti-amyloid primary antibody (monoclonal BAM10, dilution 1:1000, Sigma®) for 48 hours and then with a secondary antibody (biotinylated IgG anti-mouse, BA-9200, dilution 1:1000, Vector® Laboratories, Burlingame, USA) for 1 hour. Before revelation (VIP substrate kit for peroxidase, Vector® Labs), the reaction was amplified for 1 hour with a biotin-avidin complex (ABC Vectastain kit, Vector® Labs)^53^. For Congo red staining, sections were pretreated with 1% NaOH in 80% EthOH saturated with NaCl for 30 min. Then, they were again immersed in the same solution saturated with Congo red for 30 min. For Perls’ staining, endogenous peroxidases were first inactivated by immersion in a methanol/H_2_O_2_ solution. Then, sections were stained with 2% potassium ferrocyanide (P9387, Sigma-Aldrich®) and 2% hydrogen chloride for 20 min. Iron staining was finally intensified using DAB (1 g/l), Tris (0.2 M) and 30% H_2_O_2_ for 20 min^54^.

Microhemorrhage detection was evaluated on 20 μm thick-sections. The sections were stained by incubation, for 30 minutes at 40 °C, in a freshly prepared Perl’s reagent: potassium ferrocyanide (10%) in hydrochloric acid (20%). After 3 washings in distilled water, they were counterstained for 5 minutes in a filtered nuclear fast red solution (Vector, H-3403, Burlingame, USA).

### Correlation between *in vivo* MRI, *ex vivo* MRI and histology

MR images were manually registered to histological sections using the “3D/Volume viewer” plugin from ImageJ^55^. This plugin enables manual rotation of the 3D MR volume in any direction. We identified typical landmarks such as layers of the hippocampus, blood vessels or amyloid plaques on histological sections. The 3D MR images were then manually rotated until we could identify these landmarks in the MR images. The minimum plaque size resolvable by *in vivo* or *ex vivo* MRI was established by measuring plaque diameters on the registered BAM10 stained sections employing ZEN lite 2012 analysis software (Zeiss, Oberkochen, Germany). Freehand boundaries were drawn around the plaques and their diameter estimated from the average length of the major and minor axes from the resulting ellipsoid^18^.

The properties of the plaques detected by MRI were determined after registration between MRI and histological sections double-stained for Aβ and iron in APP_SL_/PS1_M146L_, APP/PS1_dE9_, and APP23 mice. Only cortical amyloid plaques with a diameter ≥36 µm (which corresponds to the minimum plaque size resolvable *in vivo*) were considered. A total number of 369 amyloid plaques detected on histological sections were classified into four categories. 1. Iron-positive plaques detected by MRI, 2. Iron-negative plaques detected by MRI, 3. Iron-positive plaques not detected by MRI, 4. Iron-negative plaques not detected by MRI.

### Amyloid load quantification from MR and histological sections

Amyloid load was quantified from *in vivo* MR images and histological sections of APP_SL_/PS1_M146L_ (n = 5), APP/PS1_dE9_ (n = 5), and APP23 (n = 4) mice. For MR images, cortical amyloid load was calculated by using a method similar to that reported by Jack *et al*.^39^. A total of 32 regions of interest (ROIs) per animal were analyzed for *in vivo* and *ex vivo* samples: 8 slices equally spaced along the rostro-caudal axis with 4 circular ROIs (diameter ~900 µm) drawn in the cortex on each of these slices (2 in each hemisphere). Hypointense spots were manually counted in each ROI, excluding hypointense elements that could be tracked over more than 2 adjacent slices, or that had a tube-like shape, suggesting that they were blood vessels. The area of each hypointense spot was measured in each ROI. Plaque load was determined as the ratio of the total area of hypointense spots over the area of the ROI.

For histological sections, amyloid and iron load were quantified by the same method used for quantification of amyloid load on MR images after digitization of amyloid and iron-stained sections with a Zeiss Axio Scan.Z1 (Oberkochen, Germany) whole slide imaging microscope at a lateral resolution of 0.5 μm.

## Electronic supplementary material

Supplementary Figures
