# Supplementary material for: Contrast-enhanced MR microscopy of amyloid plaques in five mouse models of amyloidosis and in human Alzheimer’s disease brains
Source: Sci Rep. 2017 Jul 10;7:4955. doi: 10.1038/s41598-017-05285-1 (PMC5504006; doi:10.1038/s41598-017-05285-1)
Supplement: Supplementary file 1 — Supplementary Figures [file 41598_2017_5285_MOESM1_ESM.pdf]

Contrast-enhanced MR microscopy of amyloid plaques in five mouse models of amyloidosis and in human Alzheimer's disease brains

Supplementary Figures

Clémence Dudeffant, Matthias Vandesquille, Kelly Herbert, Clément M. Garin, Sandro Alves, Véronique Blanchard, Emmanuel E. Comoy, Fanny Petit, Marc Dhenain

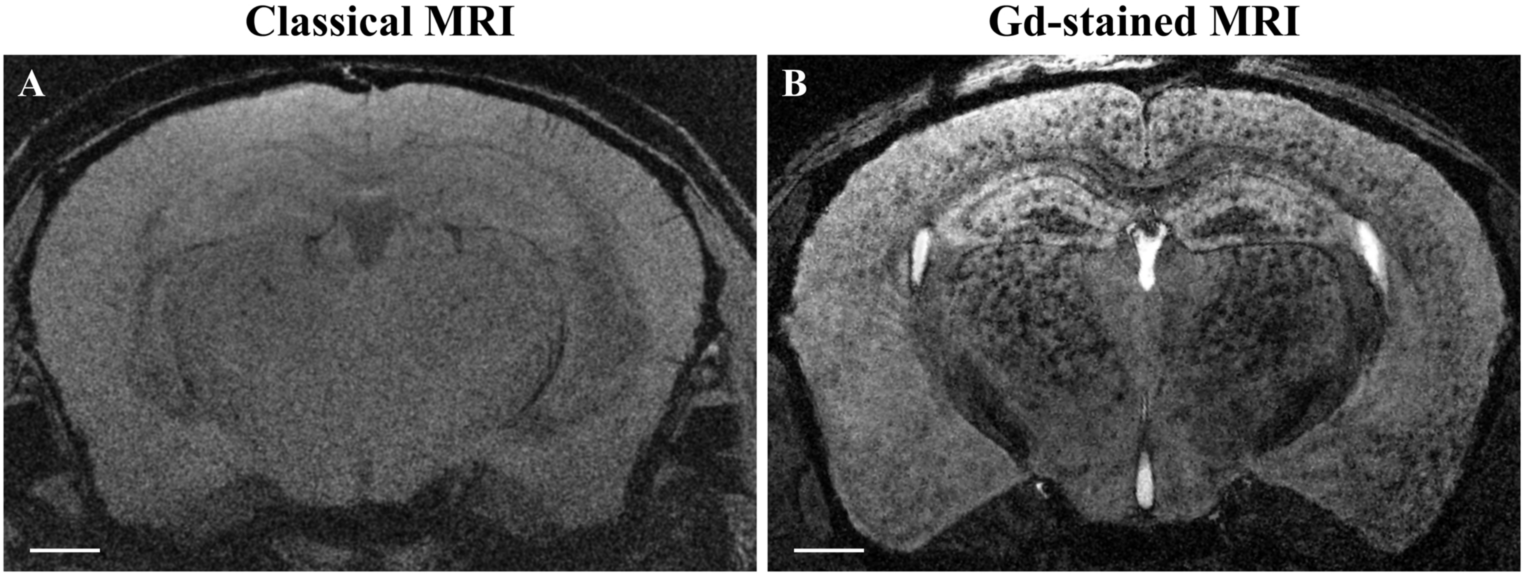

Supplementary Figure S1. MR images of an $APP_{SL}/PS1_{M146L}$ mouse before and after intracerebroventricular injection of Gd contrast agent. MR images of $APP_{SL}/PS1_{M146L}$ mice recorded without injection of Gd contrast agent had a poor signal to noise ratio and did not display any hypointense spot (A). A strong increase of the signal to noise ratio is observed in all the parenchyma after injection of Gd contrast agent and numerous hypointense spots are detected (B). Scale bar = 1 mm.

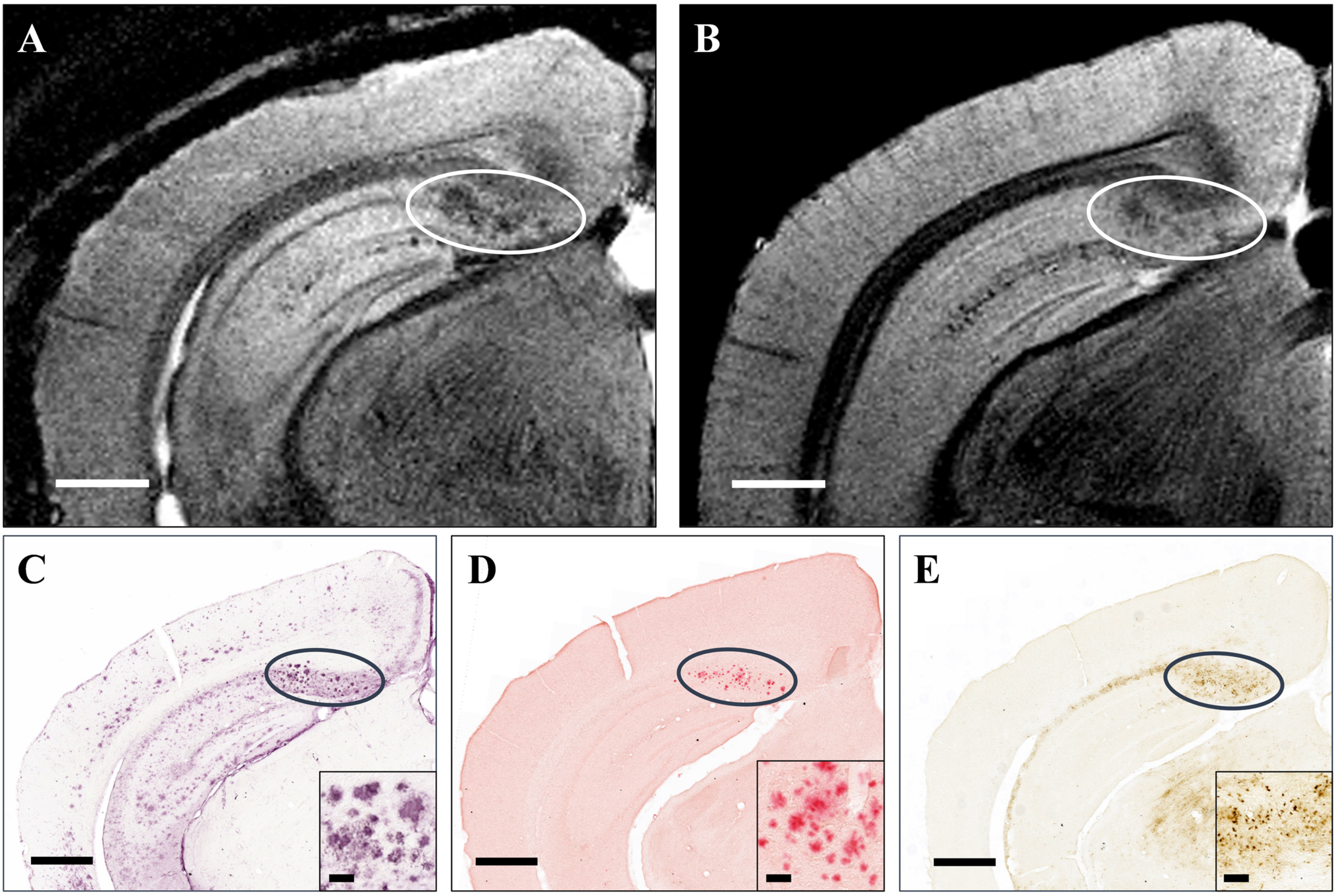

Supplementary Figure S2. Comparison between amyloid plaque detection by Gd-stained MRI and their immunohistochemistry in a 100-week-old 3xTg mouse at the level of the subiculum. Gd-stained *in vivo* (A) or *ex vivo* (B) MR images were registered to β-amyloid (BAM10, C), Congo red (D) and iron-stained (Perls-DAB, E) histological sections. Hypointense spots detected by Gd-stained MRI (A-B, white ovals) corresponding to amyloid plaques (C-D, black ovals) could only be detected in the subiculum. These subicular plaques were the only congophilic (D, black oval) and iron-positive (E, black oval) lesions observed in this strain. Plaques from other brain regions, not detected by MRI, were diffuse and iron-negative. Scale bars: 500 µm for main images and 200 µm for inserts.
